# Protocol for personal RF-EMF exposure measurement studies in 5th generation telecommunication networks

**DOI:** 10.1186/s12940-021-00719-w

**Published:** 2021-04-01

**Authors:** Maarten Velghe, Sam Aerts, Luc Martens, Wout Joseph, Arno Thielens

**Affiliations:** Department of Information Technology, Ghent University / IMEC, Technologiepark-Zwijnaarde, 126 Ghent, Belgium

**Keywords:** 5G, RF-EMF exposure, Personal measurements

## Abstract

**Background:**

The general population is exposed to Radio-Frequency Electromagnetic Fields (RF-EMFs) used by telecommunication networks. Previous studies developed methods to assess this exposure. These methods will be inadequate to accurately assess exposure in 5G technologies or other wireless technologies using adaptive antennas. This is due to the fact that 5G NR (new radio) base stations will focus actively on connected users, resulting in a high spatio-temporal variations in the RF-EMFs. This increases the measurement uncertainty in personal measurements of RF-EMF exposure. Furthermore, a user’s exposure from base stations will be dependent on the amount of data usage, adding a new component to the auto-induced exposure, which is often omitted in current studies.

**Goals:**

The objective of this paper is to develop a general study protocol for future personal RF-EMF exposure research adapted to 5G technologies. This protocol will include the assessment of auto-induced exposure of both a user’s own devices and the networks’ base stations.

**Method:**

This study draws from lessons learned from previous RF-EMF exposure research and current knowledge on 5G technologies, including studies simulating 5G NR base stations and measurements around 5G NR test sites.

**Results:**

To account for auto-induced exposure, an activity-based approach is introduced. In survey studies, an RF-EMF sensor is fixed on the participants’ mobile device(s). Based on the measured power density, GPS data and movement and proximity sensors, different activities can be clustered and the exposure during each activity is evaluated. In microenvironmental measurements, a trained researcher performs measurements in predefined microenvironments with a mobile device equipped with the RF-EMF sensor. The mobile device is programmed to repeat a sequence of data transmission scenarios (different amounts of uplink and downlink data transmissions). Based on simulations, the amount of exposure induced in the body when the user device is at a certain location relative to the body, can be evaluated.

**Conclusion:**

Our protocol addresses the main challenges to personal exposure measurement introduced by 5G NR. A systematic method to evaluate a user’s auto-induced exposure is introduced.

## Introduction

The growth of wireless telecommunication technologies has raised public concern about potential health effects of personal exposure to the radiofrequency electromagnetic fields (RF-EMFs) that are emitted by these networks and their users. In general, this exposure is divided in exposure caused by one’s own usage of mobile devices, so-called auto-induced exposure, and exposure caused by the network and other users nearby, so-called environmental exposure [7, 9, 21]. Various methods have been developed with the aim to assess this exposure such as: spatio-temporal maps [3], personal microenvironmental measurements [32, 35], spot measurements [17, 36], geospatial modelling [5, 18], survey studies [11, 37], and simulations [4, 29]. In the study of Röösli et al. [19] a protocol was developed that can reduce dependency on the used method of measurement results in different studies.

Röösli et al. [19] identified two basic types of RF-EMF exposure dosimetry studies: population surveys (using measurements) and microenvironmental measurements. In a population survey, participants selected from the general public are given a personal exposure meter (PEM) to carry with them for a certain amount of time. They are instructed to keep a diary of their activities and based on this, summary statistics on population exposure are obtained. In microenvironmental studies, a trained researcher performs the measurements in a way that represents the typical behaviour in the environment of interest. However, in both cases it has proven difficult to quantify the RF-EMF exposure induced within the user by their own devices. This auto-induced uplink (a-UL) exposure is very dependent on the location of the device relative to the body, the emitted frequency, and the power of the transmission, while the measured exposure level depends on the same factors, but has an additional dependency on the distance between the user device and the PEM. This creates a high measurement uncertainty on a-UL exposure. In microenvironmental studies this is often avoided completely by not using a personal device during measurements and limiting the research to environmental exposure. In populations surveys, the problem is often circumvented by relying on self-reported usage of personal devices obtained using questionnaires or personal usage diaries [11].

However, the up and coming 5th generation of telecommunications technologies (5G) is set out to fundamentally change the RF-EMFs the public is exposed to [2]. The methods used to measure human exposure to sources from legacy technologies (2G – 4G) will be inadequate to representatively quantify RF-EMF exposure from 5G sources [23]. This is mainly because 5G NR (new radio) base stations equipped with the enabling Massive Multiple-Input Multiple-Output (MaMIMO) technology will be able to continuously adapt their precoding to optimize the signal-to-noise ratio at the specific locations of the user devices it services [15]. This results in rapidly changing fields, both in space and time, and thus a higher measurement uncertainty. Furthermore, a person’s exposure to base stations (i.e., downlink (DL) exposure) will be much more dependent on whether they act as a user or not. Thus, a person’s auto-induced exposure will no longer be limited to the UL exposure from their own devices, but will also include DL exposure from 5G NR base stations [4]. Moreover, it is expected that the auto-induced fraction of the DL exposure will be the dominant component [4]. Therefore, the need to include the assessment of auto-induced exposure in measurement campaigns is apparent. Additionally, 5G NR networks will also use new channel access methods [29], frequency bands [16], and network architectures [31]. All these factors will alter the general public’s exposure to RF-EMFs and warrant a need for an updated protocol for measurements of personal exposure to RF -EMFs.

The objective of this paper is to develop a general study protocol for future personal RF-EMF exposure research adapted to 5G technologies. This protocol will include the assessment of auto-induced exposure of both a user’s own devices and the networks’ base stations, and will cover both survey studies as well as microenvironmental research. The proposal will be developed using lessons learned from previous RF-EMF exposure research and current knowledge on 5G technologies, including studies simulating 5G NR base stations and measurements around 5G NR test sites.

In Section 2, the necessary background information is given to understand the challenges 5G NR presents in terms of personal exposure measurements. This includes a review of the protocol currently used in personal exposure studies, an explanation of how and why the RF-EMFs in 5G NR behave differently compared to legacy technologies, and a reiteration of why a new research protocol is needed. In Section 3 describes the proposed research protocol including the necessary features of new measurement devices, the measurement procedure, and the data processing. Finally, in Section 4, we will discuss how this procedure is adapted to the challenges listed in Section 2 and what future research will be necessary to optimize the protocol.

## Background

### Review of the current exposure dosimetry protocol

As stated in the introduction, this study focuses on developing a protocol for survey studies [11] and microenvironmental studies [22] of personal exposure to RF-EMFs. Both types of research have different qualities in terms of assessing personal exposure to RF-EMFs in the population. The advantage of a population measurement survey is that the results represent human behaviour throughout full 24-h cycles [37]. The advantage of microenvironmental studies, on the other hand, is that certain exposure-related parameters are more controlled, e.g. a fixed, calibrated position of the PEM on the body and no use of own mobile devices [33].

In survey studies, the goal is to assess personal exposure in certain parts of the population [11]. These parts are typically defined by parameters such as age, living area, type of job, etc. From each group a number of participants is selected, which all are given a measurement device, such as a PEM [8, 34]. Other studies have been performed where the participants installed an application on their own smartphone, or replaced their own smartphone with an alternate that is equipped with an application, logging the transmitted power of the device and certain parameters quantifying the connectivity to the network, such as the Received Signal Strength Indicator (RSSI) [6]. The participants have to carry the measurement device (PEM or smartphone) with them for a number of days and keep a diary of their activities [34]. Furthermore, the measurement device often tracks GPS data [34]. The main requirements are that participants are randomly selected, representative for their part of the population, and that the sample of participants is large enough [11].

Currently, a-UL exposure is already the biggest source of measurement uncertainty in survey studies [37]. This uncertainty is mainly caused by a discrepancy between the measured personal exposure and the actually induced personal exposure during a-UL. An explanation for this measurement uncertainty can be found in the difference in location where the exposure is measured (location of the PEM) and where the exposure is induced (location on the human body near the user device [20]). An alternative technique to register a-UL exposure is the use of personal diaries. However, these are subject to human error, recall bias and, more importantly, the exposure is dependent of the power output of the device, which cannot be assessed by participants of a survey study. The emitted power depends (amongst other factors) on the connectivity of the phone, which cannot be deduced from measured PEM data.

In microenvironmental studies, the goal is to assess personal exposure in certain geographical areas [28]. The areas under study are divided into microenvironments. These are either smaller parts of the area defined by the typical activity performed by the public (residential, commercial, industrial, etc.), or inside buildings (offices, homes, schools, train stations, etc.), or while using a certain means of public transportation (trains, busses, etc.). The researcher then defines a path through the microenvironment or certain public transportation lines along which they perform a set of repeated measurements, potentially divided into specific timeslots, by wearing a PEM [28]. Currently microenvironmental measurements are only performed in non-user scenarios, and the measured exposure is catalogued as environmental exposure. To get a representative measurement of DL exposure in an environment, at least 15 min of walking suffices [32]. Bolte [9] determines four factors influence the measurement uncertainty of PEMs: mechanical errors, the measurement process due to hardware of software filters, the anisotropy effect, and influence of the body (shadowing, absorption, and reflection). The last three can be reduced by performing (on-body) calibrations or wearing multiple devices. Thielens et al. [26] found that the measurement uncertainty can be reduced by 2.6 dB by wearing two PEMs simultaneous and by performing an on-body calibration of the PEMs.

### Behavior of RF-EMFs in MaMIMO technologies

Throughout this paper, different sources of RF-EMF exposure are divided into five categories:

- **Environmental broadcasting downlink (BC)**: In many networks a control is sent out by the base stations to find potential users.
- **Auto-induced data transmission uplink (a-UL):** Data transmission from a user’s own device towards a base station.
- **Environmental data transmission uplink (e-UL):** Data transmission from devices of other, nearby users towards a base station.
- **Auto-induced data transmission downlink (a-DL):** In legacy technologies data transmission happens over fixed, cell-wide beams. In LTE-Advanced (4.9G) and 5G NR, narrow beams are aimed from base stations to the user device (in non-line-of-sight this results in a RF-EMF hotspot at the user device).
- **Environmental data transmission (traffic) downlink (e-DL):** Narrow beams and hotspots aimed at and around other, nearby user devices.

A non-user is exposed to the first, third and fifth categories (i.e. only environmental sources), a user is exposed to all five.

In 5G NR, downlink data transmission will occur over adaptive channels in order to maximize the signal to noise ratio at the intended user [15]. The technology enabling this is broadly referred to as (Ma)MIMO: each of the base station antenna elements will configure its phase and amplitude to ensure constructive interference at the intended user equipment (UE, e.g., a smartphone) and destructive interference at unintended users. This is also called spatial multiplexing. A user’s exposure from data transmission DL depends on the position of the UE relative to the base station: either the UE is in line-of-sight of the base station or the UE is in non-line-of-sight of the base station. In line-of-sight, the base station configuration leads to narrow beams towards the UE [29]. In non-line-of-sight, the base station configuration leads to an EMF hotspot at the UE [24]. This hotspot is created by the interference of multiple reflected and/or refracted paths. This is in contrast to legacy technologies, where data transmission DL occurs over a fixed beam (typically with an opening angle of 120°) covering a whole sector. This means that the fields transmitted by a 5G NR base station will be much more dynamic and more spatially diverse.

This also means that a big component of a user’s data transmission DL exposure will be auto-induced (a-DL exposure): by demanding data from the network, a user will pull a beam or hotspot towards their UE. The power density in this beam or hotspot will be correlated to the downlink throughput [10]. Maximum ratio transmission, the precoding algorithm used to create such RF-EMF hotspots, is only one example of the many types of precoding algorithms than can be applied on MaMIMO antennas [15]. Other precoding algorithms use destructive interference to create a zero at a user to reduce signal interference with other users. In legacy technologies, a user’s own activity has little influence on their DL exposure [35], so all DL exposure was considered environmental. The concept of a-DL exposure will be new for 5G NR.

Spatial multiplexing and focusing on users increase the signal-to-noise ratio. This increase in signal-to-noise ratio will most-likely be accompanied with higher EMF values at the user device than what can be achieved using broad beams from the base station (the current technology). This implies that a-DL exposure will likely be higher than environmental DL exposure. The increased signal-to-noise ratio also allows for the use of higher frequencies (3.5 GHz, 26 GHz and 60 GHz are candidates [25, 38]). These higher frequencies are less used in current networks because of the increased path loss and consequently low signal-to-noise ratio. However, using new precoding techniques the signal-to-noise ratio at these frequencies can be increased and more bandwidth can become available. Consequently, higher throughputs are possible.

Another factor increasing the bandwidth of 5G NR is the use of Time Division Duplexing (TDD) [4, 29]. This means that the UL and DL to one or multiple users share the same frequency resources, but are allocated different time resources. This again adds to the dynamic nature of 5G. Next to TDD, users may also be assigned separate bandwidth parts, as is currently the case for e.g. 4G, increasing the options for a base station to optimize its configuration [2].

### Implications of 5G on personal exposure assessment

With 5G NR, the importance of auto-induced exposure will increase. Currently, only auto-induced UL exposure is important, since a-DL and e-DL are the same in the current networks. However, in 5G NR, they will be separated and a-DL will have a significant impact on the user’s exposure as well, due to the beam towards or hotspot at the UE. Thus, auto-induced exposure will form a bigger fraction of one’s total exposure. In this case, the relevance of studies restricted to environmental exposure becomes questionable in the 5G era. Therefore, a protocol in which auto-induced exposure is accounted for is needed.

Next, this also leads to a higher measurement uncertainty of auto-induced exposure in survey studies specifically. We discussed the current measurement uncertainties of a-UL in section 2.1. Now, with the narrow beam or hotspot focusing, not only the a-UL, but also the a-DL exposure measurement will be highly dependent of the location of the PEM. This thus makes any measurement of auto-induced exposure much more uncertain.

Additionally, in both survey and microenvironmental studies, there will be an increased measurement uncertainty on the assessment of e-DL exposure. We speculate that many of the behaviours of e-UL exposure will also be exhibited by e-DL exposure. This is because both of them will be dependent of the proximity of other users and the amount of data transfer going to and from these users. In current microenvironmental studies, e-UL exposure shows the highest uncertainty.

Lastly, there are some causes for increased measurement uncertainty that apply to each of the five categories of exposure sources: (1) Body-shielding is one of the sources of measurement uncertainty. Due to the use of higher frequency bands, the effect of body shielding can be stronger [27]. (2) Due to the higher frequencies as well as the use of TDD, the channel bandwidths will increase. A larger bandwidth that has to be simultaneously measured by a PEM, will be accompanied by more noise. (3) Due to TDD and flexible bandwidth part configurations, transmissions can be short and potentially highly variable in the use of frequency domain and time domain resources. Since a PEM is not connected with the network, it might sample the band at the wrong time. (4) UL, data transmission DL, and broadcasting DL can all occur in the same frequency band. Therefore, using the current PEMs, it would be impossible to know what the source of the exposure is. In Section 3.2 we propose the design of a new PEM.

## Research protocol

### Activity-based exposure assessment

A user has many options on how to use the network, influencing their auto-induced exposure. Two variables are important here: (1) the location of the UE relative to the user’s body and (2) the amount of data transmission in both UL and DL cases. The location of the UE is important because the user’s exposure depends on the coupling of EM energy in the user’s body, which depends on the separation between the UE and the body. The relative position of the user’s body will also affect the channels from the base station to the UE. Therefore, it will also affect the size and shape of the beam or hotspot aimed at the UE. The DL and UL transmissions are important because a higher EM power aimed at or emitted by the UE, implies a higher exposure of the user.

Furthermore, users’ environmental exposure depends on the time and location within a microenvironment and the type of microenvironment. As an example we refer to the measurements on train rides performed in [35]: in a non-user case, the measured power density *S*_*e-UL*_ from e-UL sources during rush hours was highest on train rides, while the *S*_*e-DL*_ was lowest on train rides (no distinction was made between e-DL and BC). During rush hours (with more people on the train), the *S*_*e-UL*_ was about 12 times higher than during non-rush hours. This shows how a microenvironment and the timeslot influence environmental exposure. Now assume a user in this scenario. They undergo the same environmental exposure as a non-user and additionally the auto-induced exposure from their own use. The specific amount of auto-induced exposure is again influenced by the microenvironment: during a train ride, the user might be more inclined to use their personal device in a specific way (e.g. for streaming), causing a specific *S*_*a-DL*_ and *S*_*a-UL*_. The quality of the connectivity also affects their *S*_*a-DL*_ and *S*_*a-UL*_. Lastly, the position of their mobile device relative to their body influences *S*_*a-DL*_ and *S*_*a-UL*_ as well.

**Fig. 1:**
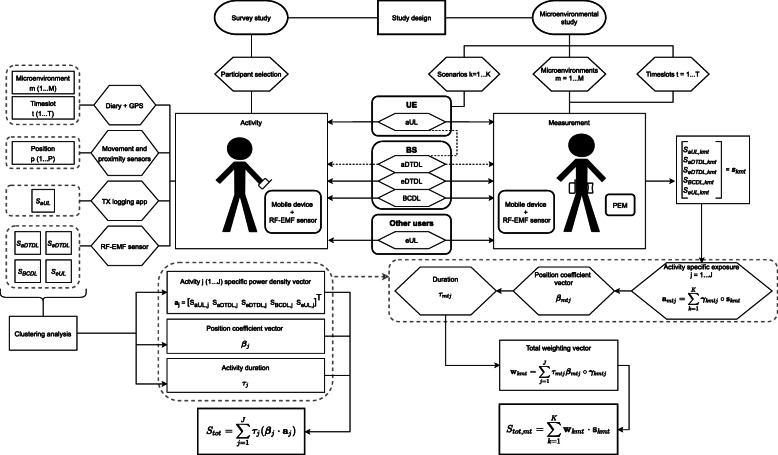
Flowchart of the measurement procedure for survey studies (left) and microenvironmental studies (right)

Therefore, we propose to move towards an activity-based exposure assessment. An activity *j* (1…J) has the following eight attributes: the microenvironment *m* (1…M), the timeslot *t* (1…T), the position of the device *p* (1…P), and the measured power densities from each of the five source categories: *S*_*a-UL*_, *S*_*a-DL*_, *S*_*e-DL*_, *S*_*BC*_, and *S*_*e-UL*_. The position *p* is the area where the UE is likely to be during activity *j* (e.g. against the air, in a handbag, etc.). Figure 1 shows a flowchart of the proposed research protocol. The study design assumes either a survey study or a microenvironmental study. The protocol is specified for both survey and microenvironmental studies.

#### Survey studies

In survey studies, exposure can be obtained directly from activities. The selected participants are given a mobile device, which tracks their GPS coordinates, timing of telecommunication-related activities, the movement and proximity of the device relative to the body, and the amount of power emitted (resulting in *S*_*a-UL*_) by the device. The device is also equipped with an (external) RF-EMF sensor measuring *S*_*a-DL*_, *S*_*e-DL*_, *S*_*BC*_, and *S*_*e-UL*_. The technical requirements for this equipment are discussed in Section 3.2. Lastly the participants can optionally track their activities in a diary. The results are then used as input to a clustering analysis [30] in order to define J activities. For each activity *j* we then define an activity specific power density vector ***a***_*j*_:

1 $$ {a}_j={\left[{S}_{aUL,j}{S}_{aDTDL,j}\ {S}_{eDTDL,j}\ {S}_{BCDL,j}\ {S}_{eUL,j}\right]}^T $$

with *S*_*source,j*_ the measured power density from the specific source category during activity j. Next, the exposure received by the user is dependent on the position *p* of the device relative to their body. Therefore we introduce a position coefficient *β*_*source,j*_ transforming the measured power density from a specific source category to the received power density on the body [12]. This is based on simulations and will be discussed in Section 3.4. This then leads to a five-dimensional position coefficient vector ***β***_*j*_ where the coefficients should be ordered in the same manner as for ***a***_*j*_ based on which source they apply to. Lastly, each activity has a duration fraction *τ*_*j*_ (duration of this activity relative to the total study duration). The total exposure based on activities can then be calculated as:

2 $$ {S}_{tot}={\sum}_{j=1}^J{\tau}_j\left({\boldsymbol{\beta}}_j.{\boldsymbol{a}}_j\right). $$

#### Microenvironmental studies

To include auto-induced exposure in microenvironmental studies, a mobile device (UE) will be needed. The UE can download and upload data during the measurement in a controlled manner, emulating a specific user activity. It is unrealistic to measure all possible activities *J*. However, it is possible to measure extreme scenarios of data transmission (maximum and minimum (while still being connected)) for both UL and DL. Together with the non-user case, this gives five data transmission situations. We listed these in Table 1 with examples of realistic data transmission scenarios at three typical positions of the UE around the body (against the ear, in front of the body carried in a hand, and in a pocket (shirt, trousers, vest, etc.)). The UE is typically against the ear when performing a phone call, which is not an extreme UL or DL data transmission case. During the measurement the researcher should keep the UE at a fixed position, from where the measured power density values can be transformed to received power density values as discussed for survey studies in Section 3.1.1. With this fixed position, five scenarios remain, each with differing amounts of UL and DL data transmission as shown in the first column of Table 1. Due to the flexible allocation of frequency and time domain resources by the base station, it is probable that the amount of DL data transmission and UL data transmission will influence the configuration of these recourses allocated to the other direction of the data stream. Assuming the a-UL allocations in (max UL) stay the same independent of the amount of a-DL and vice versa, the (max UL, min DL) situation(s) can be inferred using a linear combination of the other three situations:

3 $$ \left(\mathit{\max}\  UL,\mathit{\min}\  DL\right)=\left(\mathit{\max}\  UL,\mathit{\min}\  DL\right)-\left(\mathit{\min}\  UL,\mathit{\max}\  DL\right)+\left(\mathit{\min}\  UL,\mathit{\min}\  DL\right) $$

**Table 1:** Positions of the UE near the body in each case of the amount of data transmission in UL and DL. Some typical activities are given

Data transmission	UE locations relative to body		
	Against ear	In front	In pocket
No connection	n/a	n/a	n/a
(min UL, min DL)		Reading	No activity
(min UL, max DL)		Video streaming (DL)	Big file download
(max UL, min DL)		Video streaming (UL)	Big file upload
(max UL, max DL)		Video call	Operation requiring low latency and Cloud Computing

This results in four scenarios we propose to perform. These should be programmed in the UE to run each for a certain amount of time (e.g. 1 s) in a sequence that will be repeated throughout the measurement.

On the right side of Fig. 1 the flowchart of the proposed procedure for microenvironmental studies is shown. In each microenvironment *m* (1…M), timeslot *t* (1…T), and scenario k (1…K) we define a scenario-specific power density vector **s**_*kmt*_:

4 $$ {\mathbf{s}}_{kmt}={\left[{S}_{aUL, kmt}\ {S}_{aDTDL, kmt}\ {S}_{eDTDL, kmt}\ {S}_{BCDL, kmt}\ {S}_{eUL, kmt}\right]}^T, $$

with S_source,kmt_ the measured power density from the specific source category during scenario k, in microenvironment *m* and during timeslot *t*. These scenario-specific power density vectors can be combined using a weighted sum to estimate the total exposure per microenvironment and timeslot. From survey studies, the J relevant activities for microenvironment *m* and timeslot *t* should be selected. For each activity-specific power density vector **a**_mtj_, a linear combination of scenario-specific power density vectors with coefficients **γ**_*kmtj*_ can be made:

5 $$ {\boldsymbol{a}}_{mtj}={\sum}_{k=1}^K{\boldsymbol{\upgamma}}_{kmt j}{}^{\circ}{\boldsymbol{s}}_{kmt}, $$

with **γ**_*kmtj*_ a vector of five dimensionless coefficients (for each dimension of **s** and **a**) and ° the elementwise (or Hadamard) product. As discussed in Section 3.1.1, the total exposure is the sum of the exposure in each activity, weighted by the fraction of time spent in this activity τ_*j*_ and the position coefficient vector of each activity ***β***_*j*_. We can apply this approach per microenvironment *m* and timeslot t:

6 $$ {\mathrm{S}}_{tot, mt}={\sum}_{j=1}^J{\tau}_{mtj}\left({\boldsymbol{\beta}}_{mtj}.{\boldsymbol{a}}_{mtj}\right). $$

Equation 5 can be used to substitute ***a***_*mtj*_. This results in an expression for the total exposure based on measurement scenarios in microenvironment *m* and during timeslot t:

7 $$ {\mathrm{S}}_{tot, mt}={\sum}_{k=1}^K{\boldsymbol{w}}_{kmt}.{\boldsymbol{s}}_{kml}, $$

with ***w***_*kmt*_ the total weighting vector:

8 $$ {\boldsymbol{w}}_{kmt}={\sum}_{j=1}^J{\uptau}_{mtj}\left({\boldsymbol{\beta}}_{mtj}{}^{\circ}{\boldsymbol{\upgamma}}_{kmt j}\right). $$

### Measurement equipment

As shown in Fig. 1, a combination of two devices is proposed: (1) a personal exposure meter (PEM) and (2) a mobile device connected to the 5G NR network.

The novel PEM will be used to measure both environmental and auto-induced 5G NR exposure. In the case of TDD, it will not possible to separate UL and DL contributions by frequency alone, as all of the 5G NR signals (BC, DL, and UL) occur in the same frequency band. However, mobile network operators will synchronize 5G NR transmissions (at least per country). This means that the TDD slot format will be fixed, which, in theory, could be used to discern UL and DL exposure if the sampling speed of the PEM can be fast enough. In other words, to distinguish between at least the downlink (i.e. a-DL + e-DL + BC) and the uplink (a-UL + e-UL) exposure sources, the PEM should be able to measure the root-mean-squared power per slot of the 5G NR radio frame. For sub-6-GHz signals, the shortest slot duration is 0.25 ms (i.e. in the case of a subcarrier spacing of 60 kHz) [1]. This is much faster than any PEM today, which sample only once every 3 to 4 s. The high sampling rate will massively increase the data storage and battery life needs of the required PEM. Furthermore, it may also be possible to effectively distinguish between a-DL, e-DL, and BC, as well between a-UL and e-UL by keeping an accurate diary and additional post-processing, based on the difference in the distributions of the received powers per slot. Unfortunately, the difficulty to synchronize the PEM sampling with the specific slot timing will also induce additional measurement uncertainty.

In order to experimentally assess the exposure of a user in a 5G NR network, user equipment is needed to attract (a) beam(s)/hotspot(s). In survey studies, the mobile device should act as the participant’s own user device with which they can conduct their normal mobile activities, and in microenvironmental measurements, it will be used to emulate different scenarios. Besides the possibility of inducing a-DL and a-UL exposure, which can then be measured with the PEM, the device can be equipped with an application such as XMobiSense [14] to log the *Received Signal Strength Indicator* (RSSI) from which *S*_*BC*_ could be derived (after calibration). Equipping the mobile device with an RF-EMF sensor such as DEVIN would further enable one to keep track of a-UL exposure, which would make it easier to differentiate a-UL and e-UL. It is possible to add a PEM to survey studies as a complementary measurement device.

In the case of microenvironmental studies, the PEM(s) and the mobile device should be fixed on the body and should thus be on-body calibrated, so that the measurements by the PEM(s) can be used to estimate the shape of the hotspot or beam and help calculate *β*_*mtj*_.

In order to calculate *β*_*j*_ per activity *j* from survey studies, the location of the mobile device (the UE) relative to the body during the activity should be known. The location *p* of the UE is representative for the area where the UE can be during activity *j* (e.g. against the ear, in a handbag…). This proxy should be a worst case (i.e. as close as possible to the body) or a high percentile (e.g. the 95th percentile) of a representative set of simulations of positions in the area. The area where the mobile device is can be derived by using existing smartphone proximity sensors, gyroscopes, alternative methods of monitoring (such as motion tracking or external inertial sensors), statistics on biomechanical movements (during certain activities or in general during the day) and questionnaires or diary keeping.

### Measurement procedure

For a microenvironmental study, first, the microenvironments and timeslots to be assessed are identified. Then, the scenarios which the mobile device should cycle through are selected. Since more scenarios means less time that can be spent in each scenario, a total of four scenarios were proposed in Section 3.1.2.

In each microenvironment a measurement path is defined. Previous studies found that at least 15 min of walking along such a path obtains reproducible results within a microenvironment [28]. More scenarios will increase the measurement time that is necessary to have representable results for each scenario within the microenvironment.

A good practical measurement setup, which also reduces the measurement uncertainty, is measuring with two on-body calibrated PEMs simultaneously, such as on the left and right hips, and a mobile device on a third fixed location. The calibration procedure as lined out in [26] can be followed.

### Data processing

From the survey studies, J activities should be defined using clustering analysis. P discrete positions (locations relative to the body), microenvironments and timeslots should be defined and used as labels. Per position, the factors *β*_*j*_ should be calculated. Previously, a number of simulation studies have assessed the exposure of a body nearby a radiating EM source. Current work on numerical simulations is being done to show the shape of a local hotspot near a user, which depends on the locations of both base station and UE. Hence, by comparing the powers received by the PEMs and by the UE, and fixing the UE on a known position on the body, the shape of the local hotspot, as well as the related exposure of the body could be estimated based on those numerical simulations.

The data from the microenvironmental measurements should be weighted for each of the scenarios based on the activities present in the specific microenvironment and during the specific timeslot in order to obtain summary statistics on the exposure quantities in certain activities. As discussed in 3.2, it may be possible to split a-UL and e-UL, and a-DL, e-DL, and BC based on their different distributions. These differences in distributions are caused by the proximity of the source to the measurement device and fundamental differences in data transmission in UL, DL, and BC.

A discussion on data-cleaning (including dealing with non-detects) was already included in [19] and can be transferred to this protocol.

## Discussion

The implementation of MaMIMO base stations with adaptive precoding in the fifth generation of telecommunication networks will lead to a high spatial and temporal variability on the RF-EMFs. This will be one of the sources of an increase in measurement uncertainty on personal exposure measurements. Furthermore, MaMIMO will introduce an auto-induced downlink component to the personal RF-EMF exposure in the population. In the current protocol for personal exposure measurements, auto-induced exposure is typically omitted. These two factors present a challenge to the field of RF-EMF dosimetry. In this paper, we aim to transform this challenge into an opportunity to improve the assessment an individual’s exposure by including auto-induced exposure and by working with an activity-based model.

As on the conception of this paper, there are no large-scale roll-outs of 5G NR with millions of users, many details of the protocol suggested in this paper are still based on preliminary insights and may be subject to adaptation taking into account results from future studies. Nevertheless, based on the public concern of RF-EMF exposure, it is important to have a practical protocol ready by the time 5G NR will be rolled out.

In Eq. 1 we defined total exposure in terms of power density **a**_*j*_ during each activity j. With the evolution of user data collection, it will be possible to calculate the τ_j_’s, the fraction of time spent in each activity, for many users. The distribution of RF-EMF exposure in the population can then be assessed on an individual level, with survey studies providing **β**_j_ and **a**_*j*_. This would be extremely useful for epidemiological studies and for relating potential long term health effects to RF-EMF exposure. It is important to stress the different goals of microenvironmental and survey studies. When a systematic analysis of exposure across time, different cities and different countries is needed, e.g. in order to test the implications of certain regulatory limits or to evaluate the high daily temporal fluctuations on a train, microenvironmental measurements are preferred. When the goal is to assess the exposure distribution in a certain population group, survey studies are preferred.

This protocol encapsulates basic guidelines for the conduct of personal exposure measurements. Parts of it should be made more concrete, such as the selection of a fixed set of scenarios, or may be adapted. Other features might also be added, such as the use of this protocol to validate spatio-temporal exposure maps based on measurement nodes, base station data, and surrogate modelling [3].

This protocol can be used to measure exposure in each frequency band. The goal of the RF-EMF sensor is to measure at the UE, as such narrow beams that are aimed at the UE can be measured as well. However, the design of the RF-EMF sensor will be dependent on which frequency bands are intended to be measured.

In Table 2, an overview of the main differences and similarities between the proposed protocol and the current protocol proposed by [19] is shown.

**Table 2:** Comparison between protocols for personal RF-EMF exposure measurement studies.

	Röösli et al. [19]	This paper
Measurement device(s)	PEM	UE with sensor (+PEM)
Sample rate	3 s	0.25 ms
Calibration	Device only	Device on body
Level of analysis	Microenvironments	Activities
Technologies	2G – 4G	2G – 5G
e-BC exposure	Reduced measurement uncertainty	Reduced measurement uncertainty
e-DL exposure (in 5G)	High measurement uncertainty	Reduced measurement uncertainty
e-UL exposure	High measurement uncertainty	High measurement uncertainty
a-DL exposure (only 5G)	High measurement uncertainty	Reduced measurement uncertainty
a-UL exposure	High measurement uncertainty	Reduced measurement uncertainty

Challenges to be addressed in future work include: (1) the development of measurement equipment according to the requirements described in this paper, (2) numerical simulations validated by lab measurements in order to calculate the position coefficient vectors **β**_j_, (3) trial runs of the proposed survey study protocol in order to specify the instructions to be given to the participants and to have a data set to develop the clustering analysis of the activities, (4) trial runs of the proposed microenvironmental measurements protocol in order to define the specific parameters, such as the measurement duration, specific scenarios and their duration, and the on-body location of the measurement devices, and (5) the conversion between power density and specific absorption rate (SAR), which describes the amount of power absorbed by parts of or the whole body. The advantage of SAR is that under a-UL and a-DL exposure (from hotspots), the exposure will be limited to part of the body. This type of exposure can be more accurately described using SAR than power density. In their guidelines for RF-EMF exposure limits, ICNIRP [13] has extended the use of SAR to frequencies over 6 GHz.

## Conclusions

In this paper, the implications of the roll-out of 5G NR (new radio) on personal exposure to RF-EMFs are identified. These present challenges for personal exposure measurements. A new protocol based on the activities of users is proposed in order to overcome these challenges. This protocol includes the assessment of auto-induced exposure, which is an important part of personal exposure to RF EMFs that is currently not measured in most studies. Based on the public concern of RF-EMF exposure, it is important to have a practical protocol ready by the time 5G NR will be rolled out.
